# Model-Based Control of Observer Bias for the Analysis of Presence-Only Data in Ecology

**DOI:** 10.1371/journal.pone.0079168

**Published:** 2013-11-18

**Authors:** David I. Warton, Ian W. Renner, Daniel Ramp

**Affiliations:** 1 School of Mathematics and Statistics and Evolution & Ecology Research Centre, The University of New South Wales, New South Wales, Australia; 2 School of the Environment, University of Technology Sydney, New South Wales, Australia; University of Kent, United Kingdom

## Abstract

Presence-only data, where information is available concerning species presence but not species absence, are subject to bias due to observers being more likely to visit and record sightings at some locations than others (hereafter “observer bias”). In this paper, we describe and evaluate a model-based approach to accounting for observer bias directly – by modelling presence locations as a function of known observer bias variables (such as accessibility variables) in addition to environmental variables, then conditioning on a common level of bias to make predictions of species occurrence free of such observer bias. We implement this idea using point process models with a LASSO penalty, a new presence-only method related to maximum entropy modelling, that implicitly addresses the “pseudo-absence problem” of where to locate pseudo-absences (and how many). The proposed method of bias-correction is evaluated using systematically collected presence/absence data for 62 plant species endemic to the Blue Mountains near Sydney, Australia. It is shown that modelling and controlling for observer bias significantly improves the accuracy of predictions made using presence-only data, and usually improves predictions as compared to pseudo-absence or “inventory” methods of bias correction based on absences from non-target species. Future research will consider the potential for improving the proposed bias-correction approach by estimating the observer bias simultaneously across multiple species.

## Introduction

Often data are available giving point locations where a species is found, but no data are available concerning where a species is not found. [1] describe this as presence-only data, and examples of where such data may arise include atlases, herbarium records and species lists. Such records consist largely of incidental species sightings. When modelling the spatial distribution of a species, ideally a more reliable source of information would be presence-absence data, where sites are surveyed systematically and species recorded as present or absent. But often presence-only data are the best or only available information concerning the distribution of a species, and as such presence-only data are frequently used in species distribution modelling [2] and related applications such as wildlife fatality modelling [3]. A range of methods have been proposed for analysing such data [2], [4], [5], many of which involve generating “pseudo-absences” or “background points” to be used alongside presence points in analysis. A particularly promising method is point process modelling [6], [7], which provides a means of solving the “pseudo-absence problem” of where to choose pseudo-absence points for analysis, and choosing the spatial resolution at which to conduct analyses [6].

**Figure 1:**
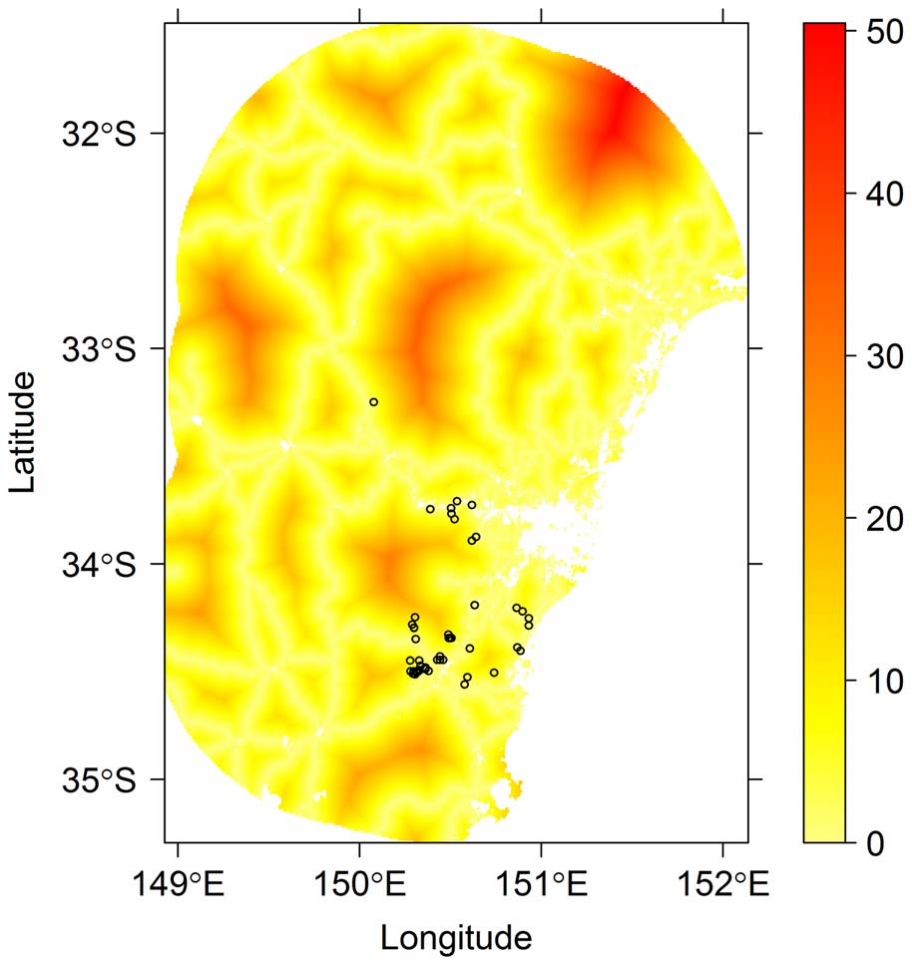
Example presence-only data. Atlas records of where the tree species *Eucalyptus apiculata* has been reported to be present, west of Sydney, Australia. These values are superimposed on a map of distance from nearest main road (in km). Note that species presences tend to be more likely to have been recorded in areas that are closer to a main road, which can be understood as a product of observer bias.

An example presence-only dataset is given in Figure 1a. This figure gives all locations where a particular tree species (*Eucalyptus apiculata*) has been incidentally reported by park rangers since 1972, in a 86,227 km^2^ area containing the Greater Blue Mountains World Heritage Area, near Sydney, Australia. We would like to use these presence points, together with maps of environmental variables, to predict the location of *E. apiculata* and how it varies as a function of explanatory variables (Figure 1). A problem doing so however is observer bias.

Inspection of Figure 1 reveals that point locations where *E. apiculata* has been recorded to be present tend to be near a major road. These presences are also frequently near Sydney, the region's major city. This observer bias is a general concern in presence-only analyses – a species is more likely to have been recorded as occurring in a place where more people are likely to see and record it. A similar issue arises in many other contexts, for example, in the recent trend towards citizen science [8], in the modelling of marine population abundance in the presence of varying catch effort [9], and in estimating treatment effects on patients in an uncontrolled observational study [10]. The problem does not arise in presence-absence analysis, because the recorded absences provide a means to control for any differences in visitation rates of different sites (by conditioning it out – we model presence/absence conditional on a site having been visited).

[11] showed across several large datasets that using presence points for non-target species as pseudo-absences can substantially improve predictive performance of single-species models. They referred to this method as using “inventory absences”, but hereafter it will be referred to as the “pseudo-absence bias correction” approach. The reason being that [12] later motivated this method of choosing pseudo-absences as a form of correction for observer bias, because if the observer bias is similar across species, such bias cancels out when looking at the presence of a species relative to other species. The implicit assumption that observer bias is similar across species usually seems plausible. In this paper, we consider two implementations of the pseudo-absence approach – (1) using point event data, for which all non-target presences are treated as pseudo-absences, and (2) aggregating presence data to grid cells, where a grid cell is treated as a pseudo-absence if it contains non-target species but lacks presence records from the target species. The point-event pseudo-absence approach has the advantage of making best use of the data available, whereas the grid-cell pseudo-absence approach is also considered because it has been proposed previously [11], [12] and is similar to what is used in Maxent [13] software.

**Figure 2:**
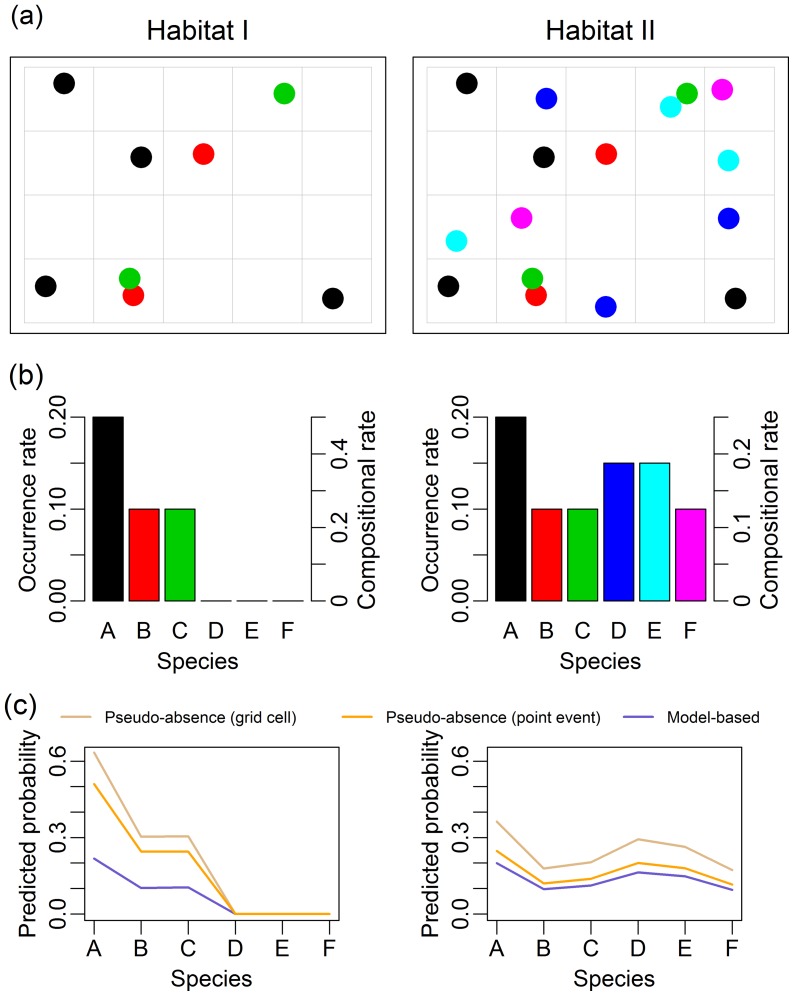
A simple demonstration of how pseudo-absence bias correction confounds the true occurrence rate of a target species with species richness. (a) Example occurrences in 20 grid cells for each of two habitat types; (b) Corresponding occurrence rates and compositional rates of occurrence in each habitat; (c) Predicted probabilities from simulation, as estimated using a model-based approach and using a pseudo-absence approach. Note that the occurrence patterns for species A–C are identical for both habitats (b), hence model-based predicted probabilities are the same for these species (c). However, the addition of species D–F at Habitat II doubled its species richness, meaning that the compositional rate halved in (b), thus pseudo-absence predicted probabilities halved in (c) *e.g.* Species A reduced from being half of all occurrences in Habitat I to being only a quarter at Habitat II, even though the absolute occurrence rate was unchanged.

While the pseudo-absence approach to bias correction has often been demonstrated to be successful at improving individual species predictions [11], [12], [14], it has one major problem – it replaces observer bias with species richness bias. This happens because the use of non-target species as pseudo-absences in effect converts the problem from estimating species occurrence to estimating species *composition* – specifically, the probability that if we encounter a species, it is the target species rather than some non-target species. This compositional rate is related to the true occurrence rate, but is confounded by non-target species richness. That is, while a species encounter is more likely to involve the target species in places where the occurrence probability is higher, it is also more likely to involve the target species at a site with less non-target species. The problem is illustrated in a hypothetical setting in Figure 2. In both Habitats I and II, the occurrence rate of species A is 20% (species A occurs in 20% of grid cells). However, in Habitat II species richness is twice as high, such that the compositional rate for species A halves (from 50% to 25%). Hence a model constructed using a pseudo-absence bias correction incorrectly concludes a halving of species A from Habitat I to II when the species is actually equally likely to be found at each site. Note that this argument applies irrespective of whether pseudo-absence bias correction is implemented using point-event or grid-cell data – simulation (Figure 2c) demonstrates that the numbers change when coarsening to grid-cell data, but the confounding effect of species richness remains.

An alternative, model-based bias correction approach is proposed in this paper, and validated against a separate systematically collected dataset. Our bias correction approach is simple to implement and intuitive – it involves modelling observer bias directly, then correcting for it when bias-free predictions are desired, and it is demonstrated to have good predictive performance.

This paper has two specific aims:

- To propose a model-based approach to bias correction, via modelling and controlling for known and quantifiable sources of observer bias, when predicting species distribution.
- To validate the proposed model-based approach to bias correction in predicting to a separate, systematically collected presence/absence dataset of 62 endemic species from the plant family Myrtaceae in a study region 86,227 km^2^ in extent near Sydney, Australia. This region includes both high-density urban areas and pristine wilderness, a substantial gradient in accessibility ideal for validation of our method.

## Model-based Bias Correction

The method proposed in this paper to deal with observer bias involves two steps: modelling the observer bias; then conditioning on a common level of observer bias at all locations to predict species distributions. This method can in principle be used together with any type of predictive model for presence-only data. In this paper, the method will be demonstrated using Poisson point process regression models [6].

Consider a predictive model for 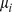, some measure of the likelihood of observing a presence for the 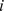
th observation used in analysis, as a function of a suite of environmental variables, stored in the vector 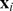 for the 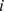
th observation. Irrespective of whether the model is constructed using a Poisson point process regression model [6], maximum entropy [13], boosted regression trees [15] or some other approach, the predictive model can be written as:
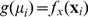
(1)where 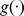 and 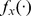 are some (possibly known) functions of 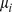 and of the environmental variables, respectively.

A key idea in this paper is to model the likelihood of observing presences 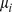 not just as a function of the environmental variables 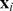, but as a function of a suite of “observer bias variables” 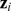 which describe how observer bias varies spatially. Hence the predictive model becomes:
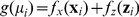
(2)


A key source of observer bias in predicting *Eucalyptus apiculata*, for example, is thought to be accessibility – hence we could use distance from the nearest main road and distance from nearest urban area as observer bias variables, which are readily calculable using standard GIS software [16].

Note that the effects of environmental and observer bias variables on 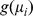 are assumed in equation (2) to be additive, *i.e.* it is assumed that there is no interaction between observer bias and the environmental effect on the target species. If this assumption is not satisfied, and hence the effect of environment on the target species changes with observer bias, then it is not possible to obtain a valid description of how the target species responds to environmental variables that is free of observer bias using *any* method.

[17] also discusses the idea of including observer bias variables in the model. Provided that the form of observer bias model 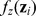 is correct, [17] explain that the precise form of environmental response 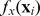 can be estimated free of bias, even if the environmental and observer bias variables are correlated. In contrast, when observer bias is ignored, unbiased estimates of species occurrence are only achievable if observer bias variables are independent of environmental variables [18]. [17] note however that the intercept term in the model is not estimable – that is, this method can only achieve a relative measure of species occurrence, not an absolute measure, unless supplemented with additional information or presence/absence data.

To control for observer bias effects in species prediction, we correct for observer bias prior to prediction. This is done by setting each observer bias variable equal to a common value (stored in 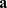, say) at all locations in the region in which predictions are to be mapped. That is, predicted values are calculated using:
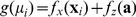
(3)


The key point is that each observer bias variable is set equal to exactly the same value everywhere in the region for which predictions are required, so that we can make predictions that correct for observer bias effects everywhere in the region. The actual values 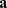 used for prediction are irrelevant, given that there is no interaction between observer and environmental variables, however some choices of common values may be easier to interpret than others. For example, in the following section, we make *Eucalyptus apiculata* predictions when distances from main road and from urban area are both taken to be zero everywhere. Subsequent predictions then have an interpretation as the likelihood of observing the species if all places had ideal access, being next to a road and an urban area.

The above proposal may be new in the context of controlling for observer bias in presence-only data, but the approach itself is quite old and widely used. It has long been used in studying the effects of one variable on another while conditioning on a covariate – for example, the classical procedure analysis of covariance, proposed over eighty years ago [19], is an application of this approach to the problem of testing for a treatment effect after controlling for the effects of some quantitative covariate. [20] used this method to control for varying survey effort in marine surveys. The idea proposed here is also related to a well-known notion in biostatistics, propensity scoring [10], long used for making causal inferences based on observational studies. The main application of propensity scoring is measuring treatment effects in a set of patients in an observational study, *i.e.* a study in which there was no opportunity to randomise the allocation of treatments to subjects.

Related but distinct methods of handling observer bias have been proposed in relation to maximum entropy estimation of presence-only data [21], [22]. [21] suggested “factoring bias out” of presence-only analyses, which is closely related to the idea proposed in this paper, except that it requires the observer bias to be known. This “bias grid” option has been incorporated into MAXENT software [22]. But a key distinction is that the MAXENT sampling grid requires the observer bias to be known *a priori*, whereas the proposal in this paper weakens this requirement such that only variables associated with observer bias need to be specified – a model is then fitted in order to use the data to estimate the observer bias. Because the observer bias is usually not known *a priori*, [21] suggested estimating it using additional data where available. [12] proposed using non-target species for this purpose, which leads to what is referred to in this paper as pseudo-absence bias correction.

## Results

We present a worked example in which we apply the model-based bias correction approach to a single species, then we evaluate the approach using 62 species and a separate presence/absence test dataset. We have written an R package called ppmlasso which can be used for Poisson point process regression with a LASSO penalty, and have included some code as Table S2 in File S1 to mimic our example analysis.

### Example application

As an illustrative example, we modelled the distribution of *Eucalyptus apiculata* (as in Figure 1) as a function of environmental variables, in a manner that controls for observer bias. This was done using a Poisson point process regression model [6], with a LASSO penalty for variable selection [23]. For details, see the Methods section. The response being modelled in a point process model is known as the “intensity”, in this case, the expected number of *Eucalyptus apiculata* presence reportings per square kilometre.

**Figure 3:**
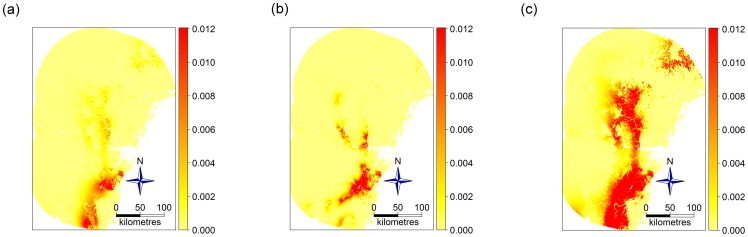
Maps of estimated intensity (in presence points per square kilometre) of *Eucalyptus apiculata* from three different models. (a) As a function of environmental variables only; (b) As a function of environmental and observer bias variables; (c) As a function of environmental variables, having modelled and conditioned on a common level of observer bias. Note that (c) predicts a higher intensity of *E. apiculata* in more remote, inland areas.

The predicted intensity of presences has been presented in three different ways in Figure 3: For a model with environmental variables only (Figure 3a) as in equation (1); For a model including observer bias variables also (Figure 3b) as in equation (2); When conditioning on a common level of observer bias (Figure 3c) as in equation (3) with 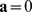, *i.e.* distance from main roads and urban areas set to zero. Note that the addition of observer bias variables to the model noticeably improved the fit – visually, the regions of higher predicted intensity (Figure 3b) better co-incide with presence locations of Figure 1, and the better fit is supported by model selection criteria (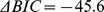). Note also that correcting for observer bias (Figure 3c) led to a qualitatively different pattern to either of the previous models, with greater predicted intensity in areas with low accessibility than either of the previous models that did not correct for observer bias (such as in Wollemi National Park, about 150km north-west of Sydney).

The LASSO model that was fitted implicitly performs variable selection, only returning non-zero coefficients for terms considered useful for predictive purposes. Non-zero coefficients were included for both observer bias variables, and as expected, the predicted intensity of *Eucalyptus apiculata* was estimated to decrease with distance from road and distance from urban area, reflecting the decreased accessibility at such locations.

### Evaluation

Our evaluation study had two goals:

1. Does model-based bias correction improve predictive performance?
2. How does the predictive performance of model-based bias correction compare to that of pseudo-absence bias correction?

We compared predictive performance on a separate presence-absence dataset, to which we applied 5-fold cross-validation, to obtain approximately independent test predictions. This is a subtle departure from the approach used in previous work [14], [24], where a separate dataset was taken “on faith” to be statistically independent of the observed presence-only data. This issue, and the precise model-fitting approach used, is considered in greater detail in the Methods section.

**Figure 4:**
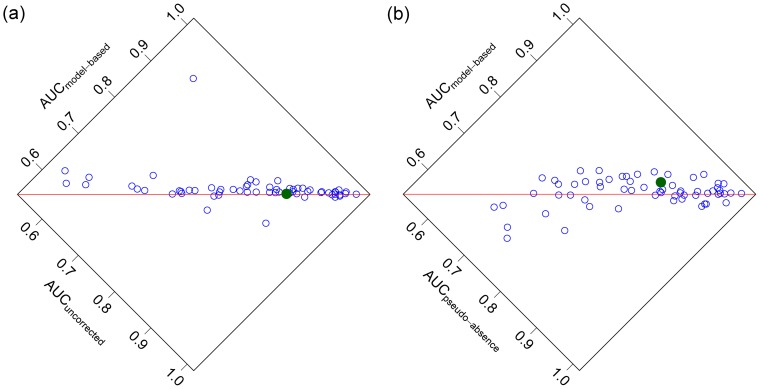
Comparison of predictive performance of different methods of correcting for observer bias. Measured as area under the ROC curve (AUC), for 62 different Myrtaceae species in the Sydney Basin. Model-based bias correction (“
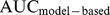
”) is compared to: (a) No bias correction (“
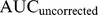
”); and (b) The pseudo-absence approach using point-event data (“
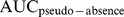
”). Note that most points lie above the line, suggesting that the model-based bias correction typically outperforms both alternative methods. The solid point on each plot represents results for the *Eucalyptus apiculata* models of Figure 3.

Key results are presented in Figure 4. In addressing the two aims of the model evaluation, it can be seen that:

1. A clear majority of species (52 of the 62) were better predicted when using model-based bias-correction than when ignoring observer bias altogether. However, the ten species for which better predictions were obtained without bias correction emphasise that the notion that we can improve predictions by correcting for observer bias is not universally true. On average, bias-correction improved predictions, but by a relatively small amount (95% CI for increase in AUC: 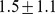
%).
2. Significantly more species were better predicted by model-based bias correction (40 vs 22) than by a pseudo-absence approach fitted to point-event data, but there were four species with generally poor model-based fits that performed substantially better under a pseudo-absence approach (AUC about 10% larger). These species dragged down the average improvement in AUC due to a model-based approach, such that it was not statistically significant (95% CI for mean AUC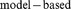-AUC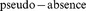: 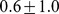
%) as compared to a pseudo-absence approach. Results were similar when using grid-cell data in place of point-event data for the pseudo-absence approach (39 vs 23 species better predicted by model-based approach, 95% CI for mean improvement 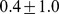
%).

The predictive performance results for *Eucalyptus apiculata*, the species of Figure 3, are presented in the plots of Figure 4 as a solid green point. For this species, the model-based correction offered little improvement as compared to using no correction, a result that can perhaps in part be attributed to the sparsity of data for this species from which to estimate observer bias.

## Discussion

A model-based bias-correction approach has been described and evaluated. This is distinct from the approaches currently used in the ecology literature [11], [12], [14], where one chooses pseudo-absence points in a selective way to reflect underlying observer bias, *i.e.* rather than modifying the model to correct for bias, the pseudo-absence approach tries to modify the data to correct for it. Model-based bias correction as proposed here, in contrast, frees us of the need to make difficult decisions concerning pseudo-absence selection, and instead puts the focus on describing the underlying processes at play using models and incorporating terms in such models to adjust for observer bias as appropriate.

The model-based approach has been demonstrated to improve predictions, and across a dataset consisting of 62 endemic species, had better performance than a pseudo-absence approach for a significant majority of species. Our evaluation gives a brief sense of some of the performance properties of the proposed method. Some other properties and potential limitations are discussed below.

A key property of the proposed approach is that the exact observer bias is not known, rather it is estimated from the pattern of the presence points in the data using a set of variables thought by the modeller to relate to observer bias. How effective this method will be in controlling observer bias will depend in part on how effective the variables chosen to model observer bias do their job, and it will depend in part on how well the effect of these observer bias variables can be estimated from the existing presence points. For a rare species in which there are few presence points in the first place, one cannot expect to reliably estimate observer bias.

One potential improvement to the approach proposed in this paper, for consideration in future work, is to use data from many species in estimating the bias-correction term. In this paper we fitted single species models, so only data from a single species was used in model-based bias correction. But it is often reasonable to assume that all species are affected by observer bias in the same way, in which case, a much better estimate of observer bias should be obtainable by jointly modelling it across all species. This would require a point process regression model fitted simultaneously across all target species, simultaneously estimating a common observer bias component, while (as in the current model) estimating a separate response to environmental variables for each species. This approach could be computationally intensive, but it would have the best of both worlds – it would share with pseudo-absence bias correction the property that data from all species would be used in estimating the bias-correction term, and would share with the model-based approach of this paper the property that it would correct for observer bias without introducing species richness bias.

A second key property to understand about the proposed approach is that its effectiveness will be reduced by correlation between observer bias variables and environmental variables [25]. This point is worthy of discussion because in most practical situations we expect some correlation between observer bias and environment – because environmental conditions affect both accessibility and where observers live. Both of these sources of correlation arose in our study region (Figure 1) – main roads tended to run along ridgetops, and people most often live on or near the coast. Hence observer bias variables were moderately correlated with elevation and thus most environmental variables. We expect such correlations to be the rule rather than the exception. Such correlation makes it more difficult (but not impossible) to tease apart environmental and observer bias effects, and subsequently we expect the proposed method to be more successful in circumstances where this correlation is weaker. Further, it is worth emphasising that it has been proven theoretically that if observer bias variables were ignored when correlated with important environmental variables, resultant estimates of species occurrence would be biased [18]. Our results lend empirical support to this result (Figure 4a).

As discussed earlier and illustrated in Figure 2, the pseudo-absence approach described in [12] can be understood as replacing observer bias with species richness bias, or at least, attempting to. Hence that method can be expected to work better when species richness is closer to uniform across a study region, and to work less effectively when there is strong spatial variation in species richness. But there was a strong species richness gradient in our data, with additional analyses suggesting species richness varied by more than a factor of ten over our study region (Figure S1 in File S1). This might in part explain the competitiveness of model-based bias correction as compared to the pseudo-absence approach to bias correction.

The proposed model-based bias correction approach can be used in combination with any predictive model capable of handling additive effects. Additivity is required such that the effects of environmental and observer bias variables can be disentangled. We used the model-based bias correction approach in combination with a point process model, a method only recently proposed for presence-only data modelling [6], [7], but a method with considerable potential, as explained in the Methods section. Whether using this modelling approach or another, it is important to consider how well suited the model is to the data at hand, and diagnostic tools have a critical role in this assessment. There is no simple answer to the question of what happens if the chosen model is not well matched to the data at hand – robustness of a model to failure of its assumptions varies with the model and with the type and extent of the violation. To some extent one can gauge the potential effects of model misspecification through measuring the predictive performance of competing models on test data, as in this paper. A quite general rule however is that if a model accurately reflects the key properties of the data, in a simple way, then the fitted model tends to have desirable properties – in interpretability as well as in predictive performance.

## Methods

### Simulation (Figure 2c)

Figure 2c reported the results of a simulation where the model-based approach and pseudo-absence bias-correction approaches were applied, in order to demonstrate how pseudo-absence approaches (whether analysing point-event or grid-cell data) measure a compositional rate rather than an absolute rate of occurrence. Details of the method of simulation are given here.

A total of one hundred random datasets were created, of the form of Figure 2a, as follows. For each of Habitats I and II, randomly located presence locations for six species (A–F) were uniformly generated across twenty grid cells such that the mean rate of occurrence per grid cell was as given in Figure 2b. Note that this is an extremely simplified setting in which there is only one environmental variable – habitat type, a binary variable – and there is no observer bias. Such a simplified setting was used to illustrate clearly the confounding with species richness that arises when using a pseudo-absence approach.

Predicted probability (/intensity) of occurrence for a species was then estimated for each dataset in one of three ways:

**model-based** Using a Poisson point process model. In this simple setting, the model fit simplifies to calculating the sample mean number of presence locations of a species per grid cell, within each habitat type.
**pseudo-absence (point event)** Using a logistic regression model, fitted to the point event data. In this simple setting, the model fit simplifies to calculating the sample proportion of point events which correspond to a species, within each habitat type.
**pseudo-absence (grid cell)** Using a logistic regression model, fitted to data aggregated to grid cells. In this simple setting, the model fit simplifies to calculating the sample proportion of non-empty grid cells containing a species, within each habitat type.

Predicted probabilities for each of the six species were averaged across the 100 simulated datasets and presented in Figure 2c.

In all simulations no LASSO penalty was used. The LASSO was not necessary since there was only one predictor variable, and using an unpenalised fit instead enabled simplification of model fit calculations.

### Presence-only data

The presence-only data used in the example application and model evaluations consist of 62 *Myrtaceae* tree and shrub species in the Greater Blue Mountains World Heritage Area (GBMWHA), west of Sydney, Australia, together with a 100 kilometre buffer zone, excluding residential areas. The spatial extent of this region is about 300×420 kilometres. The identities of the 62 species, and the number of presence records available for each, can be found Table S1 in File S1. We focussed on the *Myrtaceae* because they are a highly diverse plant family that contains many endemic species in the GBMWHA with contrasting distributions [26] – this region was declared a World Heritage Area in part because of its diversity of *Myrtaceae* species.

Presence-only points were obtained from [27] which contains both full floristic survey records and opportunistic sightings. Analyses were limited to opportunistic sightings by only using records labelled as “Default Incidental Sightings”. The full floristic survey data were kept aside as test observations to be used in evaluations.

Climate variables used in modelling (minimum temperature, maximum temperature and annual preciptation) were derived from ANUCLIM 5.1 using a 100-metre resolution digital elevation model. Fire frequency data were obtained separately from the New South Wales Office of Environment and Heritage.

### Example application

Poisson point process regression models were used because they have advantages in model specification, implementation and interpretation [6]:

- The model is scale independent, that is, doubling the number of “pseudo-absences” does not affect the final model in any way, once a sufficient number have already been included that the model has converged. In contrast, methods which instead model probabilities are sensitive to the number of pseudo-absences and/or spatial resolution [6], [17], [28].
- Implementing the model requires a set of pseudo-absences (as a device for estimation of the likelihood function via numerical integration), but the data can be queried to inform the analyst concerning the number and location of these pseudo-absences. As in [6], we chose pseudo-absences on a regular grid and used progressively finer-scale grids until the model no longer changed. The model was considered to have converged when a further doubling of the spatial resolution changed the maximised log-likelihood by less than two, and this criterion was satisfied at a a resolution of 1 km. Data can also be used to check key model assumptions, as discussed later.
- The quantity being modelled, intensity, has a natural interpretation as the expected number of presence reportings per unit area (in this case, per square kilometre).

The Poisson point process method is mathematically related to maximum entropy modelling [28], but modified to be scale-invariant and to analyse point event data rather than aggregating data to grid cells. Maximum entropy has often performed favourably in previous methodological comparisons [22], [24], and through equivalence of methods Poisson point process models inherit these advantages.

We modelled the intensity of presence points (denoted 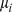 at location 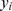) using a log-link as a quadratic function of environmental variables (although other types of environmental response, *e.g.* smoothers, could also be considered):
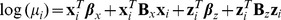
(4)where as previously 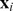 and 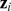 are corresponding vectors of environmental and observer bias variables, respectively. There were four environmental variables (stored in 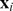) as in [6]: # fires since 1943, annual averages of maximum and minimum temperature, and precipitation. There were two observer bias variables (stored in 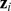) – distance from main roads and distance from urban areas, as estimated using arcGIS software [16].

**Figure 5:**
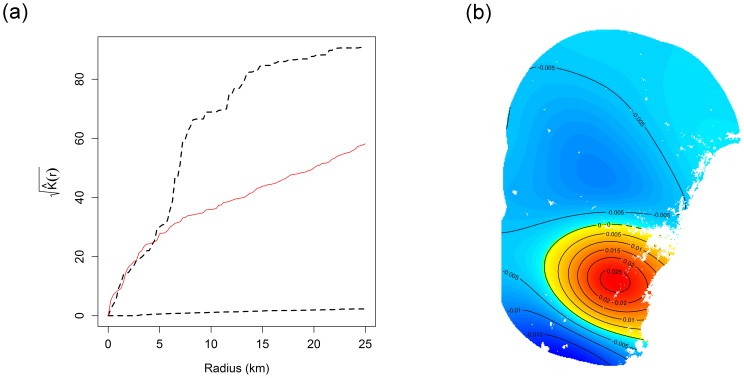
Diagnostic plots for a point process analysis of the *Eucalyptus apiculata* data. (a) Inhomogeneous 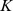-function with simulation envelope; (b) Spatially smoothed Pearson residuals. Note from (a) that the 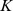 function of the observed data (solid line) runs through the centre of the simulation envelope, suggesting no evidence of inter-point dependence. Note from (b) that the spatially smoothed residual is always close to zero (always between −0.03 and 0.03), suggesting little spatial trend hence a plausible model for intensity of *E. apiculata*.

A key assumption of Poisson point process models is that the presence points are independent, conditional on environmental and observer bias variables. Goodness-of-fit diagnostic tools were used to check the independence assumption [29]. In particular, the inhomogeneous 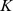-function was plotted and compared to that expected from a Poisson point process using “simulation envelopes” as in [6], [28], [30], using 100 randomly generated realisations from the fitted model. The observed data are close to the upper boundary of the envelope, marginally suggestive of a violation of the independence assumption (Figure 5a), which could potentially be handled by adding a point-interaction term to the model.

A second key assumption is that the intensity function has been accurately modelled as a function of environmental variables in equation 4. This was checked by constructing a spatially smoothed map of Pearson residuals (Figure 5b) across the study region. If there were an appreciable spatial trend in residuals over the study region, that would suggest the model for intensity had not captured some of the key structure in the data. But in Figure 5b, the mean residual was always between −0.03 and 0.03, suggesting little trend and an acceptable model fit.

Point process regression models are typically fitted via maximum likelihood [31], *i.e.* to find the parameters that maximise:
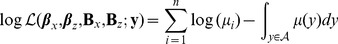
(5)where 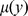 is the intensity at a location 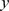, and 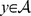 denotes all points in the study region. Note that this likelihood involves an integral, which in practice needs to be estimated using numerical integration. The “quadrature points” introduced to estimate this integral play the role of pseudo-absences [6] or MAXENT's background points [28], except that in this context we have a clear criterion to guide how these values should be added: approximation of the integral in equation (5). We added quadrature points in a regular rectangular grid at increasing spatial resolution until this integral (and hence the likelihood) converged [6]. At each step we doubled the spatial resolution, quadrupling the number of data points, and we claimed convergence when the log-likelihood changed by less than two. Plotting the maximised log-likelihood against the spatial resolution for choice of quadrature points, the likelihood appeared to have converged by about the 1
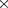
1km resolution. Fitting models at any finer spatial scale than this return equivalent maps, estimated coefficients, and standard errors [6]. We have noticed convergence at a similar spatial scale for other species also, and used this resolution in all ensuing analyses.

Rather than fitting the Poisson point process model by maximum likelihood, we included a LASSO penalty in order to automatically undertake variable selection and constrain or “regularise” parameter estimates [32]. The LASSO penalty is also used in MAXENT [13] and has been demonstrated to be a major reason for the relatively high performance of MAXENT compared to other methods [28], [33]. Whereas the MAXENT software makes an arbitrary choice of the LASSO penalty parameter [34], we estimated it by BIC, a more conventional approach which allows the parameter to be tuned to suit the data at hand [23]. In the LASSO context for point process models, BIC was defined as follows:
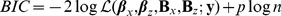
where 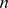 is the total number of presence locations, and 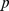 is the total number of parameters with non-zero values. We chose the value of the LASSO penalty parameter which minimised BIC.

Estimation uses the machinery of generalised linear models [35], but with observations weighted (using “quadrature weights”) in such a way that the model is scale independent [6]. The free **spatstat** software [29] on R [36] can be used for estimation, although we wrote our own code specifically adapted to SDM with a LASSO penalty, soon to be available in the **ppmlasso** package on R [36].

### Evaluation

We evaluated predictive performance of presence-only models for *Eucalyptus apiculata* and 61 other *Myrtaceae* species using data from near Sydney, Australia, as in Figure 1. Evaluations compared presence-only models to presence/absence data that were systematically collected in quadrats over the same region.

To estimate predictive performance in an unbiased fashion we require a test dataset that is statistically independent of the training dataset on which the model was originally fitted [37]. Independence is required because otherwise covariance between training and test values leads to underestimation of predictive errors (“optimism bias”), and importantly, more complex models tend to suffer greater from this issue [38], hence without correcting for this issue we might expect the predictive performance of different bias-correction techniques to be underestimated by differing amounts. [24] and others have used presence/absence data as “independent” records against which predictions from presence-only data could be tested. However, such datasets collected from the same region are not statistically independent – if the presence-only dataset had a presence record at a given location, this obviously increases the probability that a systematic transect at that location would also record a presence.

We dealt with the dependence of the validation dataset by using spatial 5-fold cross-validation: we split the study region into coarse grid cells which were 32×32 kilometres in size, randomly assigned each grid cell to one of five groups, and assessed how well a presence-only model based on four such “training” groups could predict presence/absence records in the fifth “test” group. By using coarse grid cells, there was little spatial dependence between observations across grid cells, and our validation data was closer to satisfying the important independence assumption. We repeated the process 20 times to minimise the amount of variability introduced to results via random assignment of the coarse grid cells to validation groups.

Three different approaches were compared:

**uncorrected** No bias correction: A Poisson point process regression was fitted with environmental variables only, as in equation (1).
**model-based** Model-based bias correction: A Poisson point process regression was fitted with environmental and observer bias variables, and predictions made conditioning on a common level of observer bias, as in equation (3).
**pseudo-absence** Pseudo-absence bias correction: logistic regression was fitted with environmental variables only, but the locations of presences of the 61 non-target species were used as pseudo-absence or “inventory absence” points. We considered both point-event and grid-cell data (at the 1 km resolution).

Pseudo-absence logistic regression and Poisson point process regression are closely related – they have previously been shown to be asymptotically equivalent [6], and when given two independent Poisson point processes (a marked point process with binary marks) with log-linear intensity, the model for the probability that a given point comes from one process not the other follows a logistic regression model [30]. Hence the approaches can be understood as using the same underlying model to estimate two different things – the key distinction between the above approaches is the method of adjusting for observer bias, rather than the type of model fitted.

In all cases, models were fitted using a LASSO penalty as in [28] to improve predictive performance. Such an approach is also standard in maximum entropy modelling [13]. We fitted a full regularisation path and chose the LASSO regularisation parameter using an “oracle estimator”, the optimal value for prediction to presence/absence data. This was done to reduce sampling error, as data-driven estimation of the LASSO penalty as in [28] would introduce considerable randomness to the process.

All analyses were conducted using quadrature points selected in a regular rectangular grid at the 1×1 km resolution, as previously.

Predictive performance of models was measured using area under the curve (AUC) [39] and proportion of deviance explained by a logistic regression of presence/absence data against predicted values from presence-only analyses. Both criteria returned similar results so only AUC results have been presented. Confidence intervals around estimates of average difference in AUC were constructed using a paired 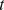 approach.

## Supporting Information

File S1 **Appendix.**
(PDF)
