# Supplementary material for: Model-Based Control of Observer Bias for the Analysis of Presence-Only Data in Ecology
Source: PLoS One. 2013 Nov 18;8(11):e79168. doi: 10.1371/journal.pone.0079168 (PMC3832482; doi:10.1371/journal.pone.0079168)
Supplement: File S1 — Appendix. (PDF) [file pone.0079168.s001.pdf]

File S1 – Appendix

Model-based control of observer bias for the analysis of presence-only data in ecology

David I. Warton, Ian W. Renner and Daniel Ramp

Table S1: Species list.

Species Name	# Presences	Species Name	# Presences
Angophora bakeri	102	Eucalyptus cunninghamii	40
Angophora costata	366	Eucalyptus deanei	73
Angophora hispida	104	Eucalyptus glaucina	66
Angophora inopina	118	Eucalyptus gregsoniana	33
Austromyrtus tenuifolia	26	Eucalyptus haemastoma	134
Backhousia myrtifolia	119	Eucalyptus hypostomatica	25
Baeckea brevifolia	26	Eucalyptus longifolia	26
Baeckea diosmifolia	59	Eucalyptus luehmanniana	107
Baeckea imbricata	22	Eucalyptus macarthurii	36
Baeckea linifolia	39	Eucalyptus paniculata	93
Callistemon citrinus	105	Eucalyptus parramattensis	125
Callistemon linearifolius	53	Eucalyptus piperita	243
Callistemon linearis	89	Eucalyptus praecox	21
Callistemon rigidus	23	Eucalyptus pulverulenta	37
Callistemon shiressii	78	Eucalyptus punctata	404
Corymbia eximia	95	Eucalyptus rossii	90
Darwinia biflora	238	Eucalyptus sparsifolia	130
Darwinia diminuta	35	Eucalyptus squamosa	32
Darwinia fascicularis	40	Eucalyptus sturgissiana	48
Darwinia grandiflora	48	Kunzea capitata	72
Darwinia peduncularis	44	Leptospermum arachnoides	72
Darwinia procera	71	Leptospermum epacridoideum	40
Eucalyptus agglomerata	60	Leptospermum parvifolium	60
Eucalyptus amplifolia	65	Leptospermum squarrosum	51
Eucalyptus apiculata	54	Melaleuca biconvexa	379
Eucalyptus benthamii	45	Melaleuca deanei	111
Eucalyptus burgessiana	44	Melaleuca decora	117
Eucalyptus camfieldii	88	Melaleuca styphelioides	114
Eucalyptus canaliculata	28	Micromyrtus blakelyi	50
Eucalyptus cannonii	93	Syzygium paniculatum	83
Eucalyptus capitellata	80	Tristaniopsis collina	29

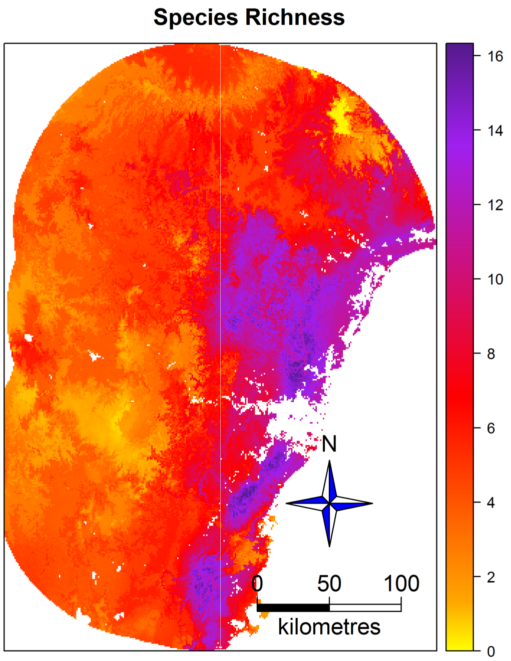

Figure S1: *Myrtaceae* species richness in the Blue Mountains region. Species richness has been estimated as the sum of predicted probability of occurrence across the 62 species, from models fitted to the presence/absence data of 8,678 survey quadrats. Models used quadratic logistic regression models with LASSO penalties (as elsewhere in the manuscript).

Table S2: R Code

```
library(ppmlasso)

data(BlueMountains)


# Pre-standardise observer bias variables

backg.env = BlueMountains$env

stand.D_MAIN_RDS = standardise.X(backg.env$D_MAIN_RDS)$X

backg.env$D_MAIN_RDS = stand.D_MAIN_RDS


# To fit a Poisson point process model at a spatial resolution of 1km

ppmForm = ~ poly(TMP_MIN, RAIN_ANN, degree = 2) + poly(D_MAIN_RDS, degree = 2)

ppmFit  = ppmlasso(ppmForm, sp.xy = BlueMountains$eucalypt,

                            env.grid = backg.env, sp.scale = 1)


# Model actually used in example analysis had the following formula:

# ppmForm = ~ poly(FC, TMP_MIN, TMP_MAX, RAIN_ANN, degree = 2)

#                                    + poly(D_MAIN_RDS, D_URBAN, degree = 2)


# To predict using model-based control of observer bias (at min value for D_MAIN_RDS):

newEnv = BlueMountains$env

newEnv$D_MAIN_RDS = min(stand.D_MAIN_RDS)

pred.biasCorrect = predict(ppmFit, newdata=newEnv)


# To find the resolution (in the range from 0.5 to 16 km):

scales = c(0.5, 1, 2, 4, 8, 16)

findres(scales, sp.xy = BlueMountains$eucalypt,

                      env.grid = BlueMountains$env, formula = ppmForm)

#which returns the log-likelihood at each scale, difference < 2 at 1km scale


# Diagnostic plots as in Fig 5:

kenv = envelope(ppmFit, fun = Kinhom)

resid.plot = diagnose(ppmFit, which = "smooth", type = "Pearson")
```
